# On Burns and Scalds

**Published:** 1807-01

**Authors:** 


### Medical and Phyfical Journal.

VOL. XVII.] JANUARY, 1807. [NO. 95.

*Printed for R. PHILLIPS, by W. Thorne, Red Lion Court, Fleet Street, London.*

### *To the Editors of the Medical and Phyfical Journal.*

GENTLEMEN,

I Am happy to see the treatment of Burns and Scalds under investigation; for, in a country so rich in mines and manufactories, productive of great and frequent danger from fire, it cannot fail to be both useful and interesting.

The discussion has been principally undertaken by Dr. Kentish and Dr. Kinglake, between whom there seems a perfect opposition in theory and practice. A demonstration of the truth would, probably, reconcile their opinions and establish a certain practice, and it is my wish to contribute towards these desirable ends. My experience in burns and scalds has been limited; but such as it is, you shall have it, and the reflections thence arising. I believe that both the Doctors will be found to use excellent remedies; but that the period of their application will constitute the chief object of enquiry, and that, in this respect, both the Doctors will re-consider their practice.

During the last campaign in Holland, I burnt the tips of eight fingers with the contents of a phosphoric phial, which exploded, and settled on them. The pain was excessive, and I had no solvent for the phosphorus at hand. I dipped my hands in cold water, which immediately removed the pain; but it recurred with equal severity as at first, the moment they were withdrawn, or the water became warm. It was remarked, both by myself and others, that the water became sooner heated than it would have done in a kettle on the fire. In this case, water was adequate to the removal of pain; but as the burning cause adhered to the fingers, and re-acted so soon as the water was removed, it necessarily failed of curing. Some years afterwards, in making the nitrous oxyde, my retort, containing three ounces of boiling nitrate of ammonia, and of course, at about 500 degrees in temperature, burst, and diffused the salt over the back of my hand. The salt had just began to chrystalize, and consequently to give out additional heat, when I plunged my hand into the water of my chemical bath. Never was there a rapider transition from torture to delight. These sensations I could alternate at pleasure for a considerable time afterwards by simply withdrawing my hand from the water. Having a party with me, and being unwilling to neglect them, I got a sponge full of brandy, as more evaporable and cooling than water, and kept it applied to my hand, till it was cured. This was accomplished in four hours without even vesication. When the sense of heat arose, the sponge was dipped in cold brandy, till it ceased, not to recommence. I own the brandy was here applied as likely to act similarly to the spirit of turpentine, without its odour; but I had no sensation from it, different from that of cold water.

Since then, I have applied a bit of rag, or blotting paper, to slightly scalded or burned parts, and have poured spirits upon them, passing over them a current of air from a small tube, till the sense of freezing arose. This sensation requires to be produced two or three times, before a cure is effected, which even, where the skin is cauterised by hot iron, may be expected in ten minutes.

I have seen a square inch of skin that was burned to the appearance of charring and surrounded by inflammation, perfectly cured by this simple process, in ten minutes. No sloughing nor ulceration took place, but the crust came off dry, and left a sound surface.

About four years since, while I happened to be in London, I was consulted by an ingenious relation in the neighbourhood of Edinburgh, whose son having been in the ash pit of a large boiler when it burst, was overwhelmed by the boiling water. Only a small part of his face escaped. The father, obeying an instinctive impulse, plunged his boy into the adjoining stream, there retained him till he complained of cold, and then stripped and put him to bed.

I cannot find this gentleman's first letter to me, which stated, correctly, as I remember, the symptons which might in such a case be expected. They were, shiverings, a general sense of cold, and a quick, low, feverish pulse, &c. But he says, in his second letter, " Upon the third day after after the accident, we sent for a Mr. Cleghorn, who takes the most dangerous cases from the Medical Gentlemen. He sat four hours dressing the boy with the only articles he uses—vinegar and chalk. He then told me, he never had seen such a dreadful scald, and that the boy could not live, desiring that we should send for Mr. Curtis, to whom he had communicated the virtues of the chalk and vinegar. Mr. Curtis, however, had not so much faith, and the vinegar was only applied where there were no wounds. These they dusted with chalk, and covered with cloths spread with lime-water and oil. In this way we went on, dressing, till yours arrived.

" Mr. Astley, and two other Medical Gentlemen were here at the time. I gave them your letter. They all agreed that your mode was infinitely superior to ours.

" The whole of the burning pain we had destroyed in the course of the first night, with spirits; for after that, neither warm cloths, nor the hot bricks kept at his feet gave him any pain. The moment that was the case, the Medical Gentlemen said, the ointments, &c. &c. recommended by you, should have been applied. Some they made, others they sent for; and several of the slightest scalds have already got skin; and the last two nights John has slept quite well. This letter was dated the 14th of December 1802.

" The next was dated the 22d of April, 1803, and says, I am the most indolent correspondent alive, or I would have written to you long ago, and thanked you in the kindest manner for your excellent advice and attention to John—for when there were not two square inches of skin on any one part of his body, had he not been treated in the best manner, he must have gone home; and now been singing with John Cabecea, of whom the Lord said unto the Angels; Silence, ye calves, and let me hear John Cabecea sing.—He is now quite well; but a little marked on one side his face."

I look upon this case as valuable, because it is delivered by a gentleman of sound judgment, unbiassed by professional prejudice; and because it goes some length to prove, that both Dr. Kentish and Dr. Kinglake, may be right in their treatment of scalds, except as to the period of its employment.

According to Dr. Kentish, the cold water ought to have killed the patient; and according to Dr. Kinglake, the turpentine ointment, opium, and wine, ought not to have been survived. It appears, however, from the above case, that had the stimulant practice immediately succeeded the cooling, the most fortunate event might have been expected. The cooling treatment must have had an excellent effect, when it removed all the burning pain in the course of the first night; the stimulant an admirable effect, when it saved the boy's life. I shall endeavour to reconcile these jarring doctrines, of which we have so many in medicine. In scalding, some hot fluid is applied to the skin; acute and violent pain is the consequence, with a sensation of pungent heat, redness, vesication, ulceration, mortification. Such is the natural course, except that mortification does not always follow.

Immerse the skin in water, or evaporate upon it that or any of the evaporable fluids, and none of these troublesome symptoms arise. The first thing observed, is the abatement or removal of pain, and of the sensation of heat. Withdraw the part scalded from the cooling process, and the pain with the sensation of heat recur. Were the sensation of heat, which seems to create that of pain, derived from the heat of the scalding fluid alone, it ought never to return, if once banished. It must, therefore, arise from the part scalded, when it occurs a second time. In proof of this, a scalded hand would heat water nearly as quickly as fire would, which the heat imparted to the hand by the scalding medium would not do. There is then excited in the scalded part, an action tending to the evolution of farther heat, which heat is sufficient to disorganize the subjacent parts. This must be true, because, in proportion to the rapidity with which you remove the heat of the part, you diminish the pain, the index of inflammatory disorganization. There is a violent stimulus, heat, applied to the skin, producing, according to Dr. Kentish's theory, or Dr. Brown's, excessive excitement: how then is this excitement to be diminished, but by the subduction of stimulant power? What can remove the stimulus itself, but water or cold in some form? In slight cases, when the pain and heat are removed, so as not to return when the cooling plan is suspended, cure is accomplished.

As a farther support to this reasoning, I am assured by a surgeon of this place, who sees a large proportion of the accidents from the explosions of hydrogen in the neighbouring coal mines, that in cases where the turpentine remedies were applied immediately, the patients suffered more, and were a fortnight longer in recovering, than in those where they were deferred till his arrival in the country.

try. I knew a case, many years ago, exactly parallel to that of the boy's above mentioned.

He was not plunged into water, but carried home and treated with lime-water and oil, the practice of the day, and he suffered dreadful pain and ulceration, and with great difficulty recovered in not less than twelve months. In slight cases then, it would seem that no practice can be better than what has been mentioned, namely, the most rapid cooling. Water is always at hand, and by having currents of air from bellows or tubes passed over it, even if little be procurable, may probably answer every purpose. Spirit of wine or æther might be preferable. If this be reasonable doctrine, it goes so far in support of Dr. Kinglake's.

But from the case of the boy, it will appear, that on the removal of the pain and sensation of heat, there is a tendency to low fever and general debility, and hence, probably, to mortification. In that case surely, sound practice, if Dr. Kentish had never written his valuable book, would direct to the external and internal use of stimuli.

But as it is known, that in proportion to the suddenness and violence of the application of stimuli to the human body, so is the consequent exhaustion of the vital principle or excitability; it is fair to expect that where actual fire, as in the explosion of mines, has been generally applied, the exhaustion will be sudden and great. Such a state may take place in that case, as did not occur in the boy before-mentioned, till after the cooling process.— Surely then we ought to take the benefit of Dr. Kentish's excellent practice, which, in all probability, saved that boy's life.

Upon the whole, it would seem that while sensation of heat and pain exist, the cooling treatment is proper; but when these are removed, and symptoms of debility occur, or when they primarily appear, the stimulant plan ought to be adopted.

*Nov.* 25, 1806. T. M. W. W. M. D.

